# Clinical significance and management of meniscal extrusion in different knee pathologies: a comprehensive review of the literature and treatment algorithm

**DOI:** 10.1186/s43019-022-00163-1

**Published:** 2022-07-18

**Authors:** Konstantinos G. Makiev, Ioannis S. Vasios, Paraskevas Georgoulas, Konstantinos Tilkeridis, Georgios Drosos, Athanasios Ververidis

**Affiliations:** 1 Orthopaedics, University General Hospital of Alexandroupolis, St. Niarhos 1, Dragana, 68100 Alexandroupolis, Greece; 2 Orthopaedics, Democritus University of Thrace, University General Hospital of Alexandroupolis, Alexandroupolis, Greece

**Keywords:** Meniscal extrusion, Centralization, Knee osteoarthritis, Root tears, Anterior cruciate ligament reconstruction (ACLR), Meniscal allograft transplantation (MAT)

## Abstract

The menisci are crescent-shaped, fibrocartilaginous structures that play a crucial role in the load transition and distribution of the contact forces along the tibiofemoral articulation. Meniscal extrusion (ME) is a radiological finding, especially in magnetic resonance imaging (MRI) scans, for which there has been growing interest in recent years. ME, in the coronary plane, is defined as the maximum distance of the most distal end of the meniscus from the border of the tibial plateau, where the tibial eminences are the most prominent, without taking into account the osteophytes. Although there is still controversy in the literature in respect of the optimal cutoff value, a threshold of 3 mm is considered significant. ME has no specific clinical finding or sign and it is encountered in many knee pathologies. It is associated with either rapidly progressive knee osteoarthritis or early onset of knee osteoarthritis and increased morbidity. In this review, we delineate the clinical significance of ME in various knee pathologies, as well as when, why and how it should be managed. To the best of our knowledge, this is the first study to elaborate on these topics.

## Introduction

The menisci are crescent-shaped, fibrocartilaginous structures located between femoral and tibial condyles, which are important for load transmission and distribution, shock absorption, secondary stability and proprioception of the knee joint [1]. The medial meniscus covers 50–60% of the medial tibial plateau, and the lateral meniscus covers 70–80% of the lateral tibial plateau. Meniscal injury decreases their functional properties, resulting in degeneration of the knee joint [2]. However, it is still controversial whether meniscal pathology leads to osteoarthritis (OA), or vice versa. Meniscal tears are grossly divided into degenerative and traumatic [3]. Degenerative tears occur mainly in the medial meniscus and are most commonly encountered in older patients without any history of significant trauma [4]. On the contrary, traumatic tears usually involve the lateral meniscus after an acute traumatic episode with concomitant ligamentous injury, especially anterior cruciate ligament tear, in younger patients [5]. Most of the meniscus pathologies have recently been correlated with meniscal extrusion [3, 6].

**Fig. 1:**
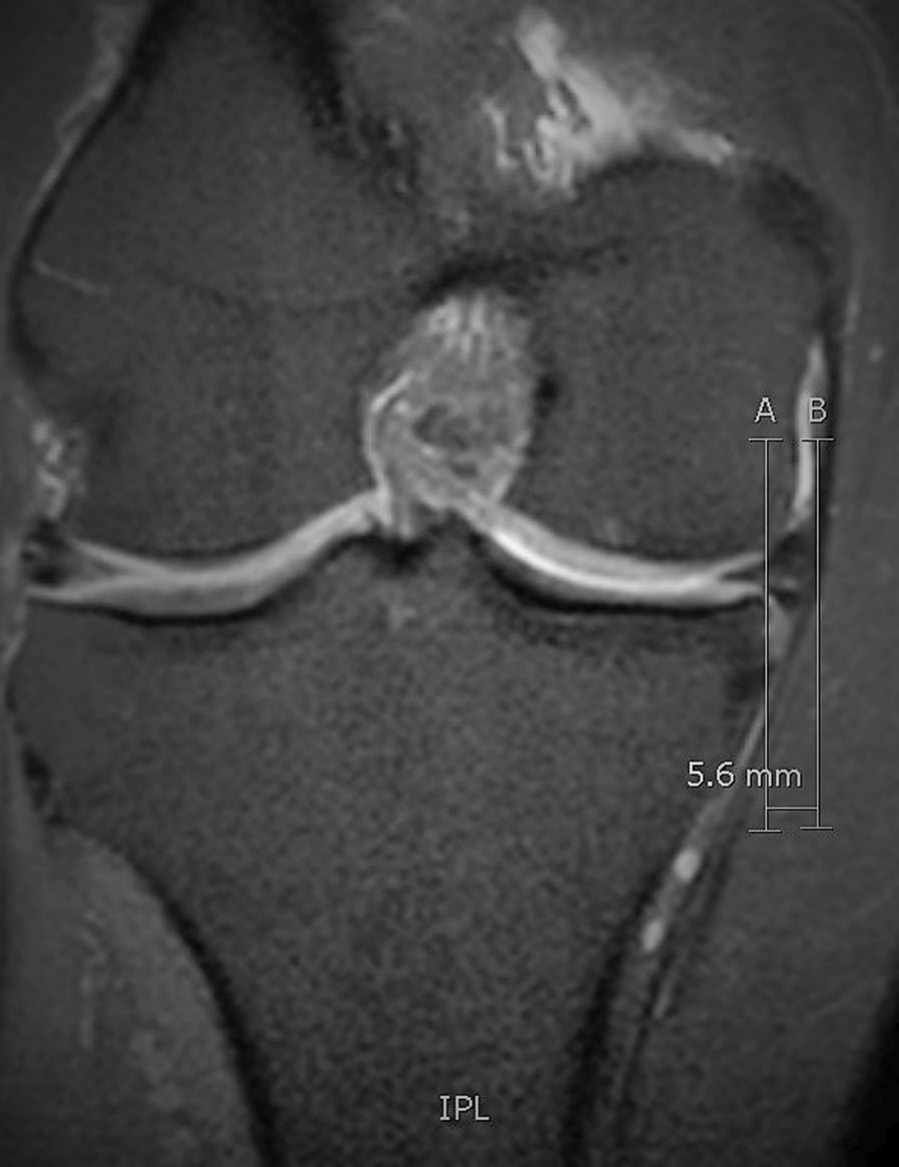
Magnetic resonance imaging (MRI) mid-coronal view of the right knee, where the tibial eminences are most prominent. Medial meniscal extrusion (MME) is measured 5.6 mm

The term meniscal extrusion (ME) is used to describe the radial meniscal body displacement beyond the peripheral tibial plateau margin [7, 8]. A threshold of 3 mm, which was initially introduced, is most commonly used to define a significant ME [8]. Nevertheless, there is still controversy in the literature regarding the optimal threshold, as a few studies have proposed a value lower than 3 mm [9, 10], whereas other researchers suggest that a cutoff value higher than 3 mm is optimal [11–13]. Moreover, instead of a single value, Miller et al. defined significant ME as when more than 25% of the meniscal body width extends beyond the tibial limit [14–17]. Magnetic resonance imaging (MRI) is the method of choice for the detection and evaluation of ME (Fig. 1), although it may underestimate ME, as the assessment is routinely not performed under weight-bearing conditions [18–20]. Therefore, ultrasound (U/S) was recently introduced as a reliable alternative to MRI for the evaluation of ME, which also offers the possibility for dynamically assessing ME in various positions, as well as in a weight-bearing state, in an inexpensive and feasible manner [20–22]. Interest in ME has grown exponentially in recent years, as it is associated with several pathologies, such as knee OA, meniscal posterior root tears (MPRT), post-anterior cruciate ligament reconstruction (ACLR) and post-meniscal allograft transplantation (MAT) [6, 23, 24]. More importantly, it leads to rapid progression of the degenerative process of the knee as well as substantial morbidity and pain [25–28]. However, knowledge regarding the management of ME remains limited. Therefore, with this article we aim to provide an up-to-date review of the literature with an emphasis on the clinical significance and new concepts in the treatment and reduction of ME in the different knee pathologies in which it is encountered.

## Knee pathologies and ME

### Osteoarthritis

ME can occur in knee OA [8, 10], and its presence is significantly correlated with increased articular cartilage loss in the tibiofemoral joint and osteophyte formation [29, 30]. Hart et al. [31] showed that lateral ME was associated with worsening of patellofemoral joint OA features at 2-year follow-up. Several studies also argue that the presence of ME is predictive of knee OA progression [32–34]. Results regarding the correlation of ME with pain in knee OA remain controversial. While many studies have shown a positive association [35–38], other studies investigating the association between ultrasound features, including ME, and pain in a series of osteoarthritic knees found no correlation between pathological ME (> 3 mm) and pain in knee OA. [39–41]. Kijima et al. [42] found that in knees with the same grade of knee OA, extrusion of the medial meniscus was greater in patients who experienced pain in comparison with painless knee OA. They then measured the medial meniscal extrusion (MME) with U/S under weight-bearing and reported a cutoff value of MME for knee pain of 4.3 mm, with sensitivity of 84% and specificity of 85% [43]. In addition, given that the presence of severe ME promotes rapid progression of knee OA [44, 45], the importance of inhibiting this process and delaying the inevitable total knee replacement (TKR) is critical.

Conservative treatment constitutes the first line of therapy in mild or moderate knee OA [46, 47]. Treatment with glucosamine, chondroitin sulfate and licofelone has been found beneficial in patients with knee OA and ME, as it may lead to alleviation of symptoms and reduce cartilage volume loss over time. However, it remains unknown whether it is capable of delaying or modifying OA progression, and further research is warranted [32, 48, 49]. Lateral wedge insoles have been found to reduce MME and could also supplement a conservative treatment of knee OA, as they could potentially delay the degenerative process [50]. However, arthroscopic debridement and osteotomies around the knee are also being employed in some instances in moderate knee OA before a TKR is required [51, 52]. Wang et al. [53], in a retrospective review of 131 patients who had undergone arthroscopic surgery for knee OA and were followed up for 4 years, found that in regard to pain relief, arthroscopic surgery was also beneficial in patients with major MME (> 3 mm). Goto et al. [54] showed that varus alignment factors, and specifically medial proximal tibial angle (MPTA), were significantly related to the extent of MME, especially as the knee OA grade progressed. Therefore, they suggested that this type of OA patient, with MME and varus malalignment with a low MPTA, could benefit primarily from an early intervention with high tibial osteotomy (HTO), thus delaying the progression of knee OA. Astur et al. [55] verified this hypothesis by performing 66 HTO, where they measured ME with MRI preoperatively and postoperatively, and found that at 6 weeks postoperatively, the ME had decreased from a mean of 3.9 ± 0.6 mm to a mean of 0.9 ± 0.5 mm. Furthermore, at 2-year follow-up, patients experienced significantly less pain, with improved clinical outcomes.

### Meniscal posterior root tear—meniscotibial ligament lesions

ME is strongly correlated with posterior root tears of the menisci [6]. MPRT are defined as an avulsion fracture or a radial lesion occurring within 1 cm of the bony tibial attachment [56]. This pattern completely disrupts the continuity of the circumferential fibers, leading to the loss of the hoop tension and substantial ME, thus promoting rapid progression of knee OA [1, 57]. Management of the MPRT consists of nonoperative treatment, partial meniscectomy and root repair, and primarily aims to prevent the development of OA [58]. Nonoperative treatment is associated with poor clinical outcomes, especially in the presence of a large ME [59], and exacerbation of OA with a higher incidence of subsequent arthroplasty over 5 years of follow-up [60]. Therefore, nonoperative treatment should be reserved for nonsurgical candidates [61].

Historically, MPRT were treated with partial meniscectomy. However, several studies showed that partial meniscectomy, particularly when the ME was profound [62, 63], failed to prevent the progression of OA, as it creates a meniscus-deficient state, and leads to significant functional impairment with high rates of arthroplasty [24, 60]. Consequently, partial meniscectomy is only utilized for symptomatic MPRT (catching/locking) when a root repair, due to excessive degenerative changes and poor quality of meniscal tissue, is contraindicated [61, 64].

Currently, MPRT repair constitutes the treatment of choice, since it restores the hoop tension and the joint kinematics [65], halts the progression of OA and lowers the rate of arthroplasty [66, 67]. Root repair is indicated when varus malalignment is minimal (< 5°), the cartilage wear is not advanced (Outerbridge grade 1–2) and it can be performed arthroscopically either with a transtibial pullout technique or with suture anchors, each with its respective advantages and disadvantages [58]. Although meta-analyses validate the superiority of root repair over partial meniscectomy in terms of clinical outcomes, they also highlight the inability of root repair to successfully reduce ME [68, 69]. One way to reduce postoperative ME is by early intervention, specifically within 3 months after the MPRT [70–72]. However, it has also been hypothesized that MPRT is not the sole contributing factor predisposing to ME. Also taking into consideration that residual ME after MPRT repair correlates with worse outcomes regarding both clinical parameters and OA progression [72, 73], a better understanding of the causality of ME is needed in order to appropriately address it.

**Fig. 2:**
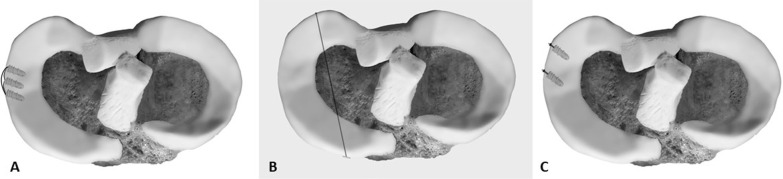
**a** Meniscotibial ligament repair with three interconnected suture anchors, **b** centralization with a transtibial tunnel or **c** with isolated suture anchors (usually with two or three suture anchors)

Current research has directed its focus to meniscotibial ligaments (MTL), which stabilize on the tibial plateau and centralize the meniscus, as an additional potential causative factor for ME. Krych et al. [74] retrospectively analyzed MRI series with isolated ME without concomitant serious knee pathologies, and found that isolated significant ME (> 3 mm) was associated with MTL abnormalities. In another study where they examined MRI series both before and after medial MPRT, they showed that MTL disruption and ME predated the MPRT, thus emphasizing the potential role that MTL could play as a causal factor of ME, and even suggesting that disruption of MTL should be addressed alongside MPRT repair in order to reduce ME [75]. Debieux et al. [13] proved biomechanically that ME is possible with intact meniscal roots and that centralization of the meniscus restores the tibiofemoral contact pressures back to normal. In a biomechanical study, Paletta et at. [76] showed that induction of MTL lesions in cadaveric knees resulted in ME after only a few cycles of loading and that repair of the lesion led to a significant reduction of ME. Furthermore, they performed an open MTL repair in 15 patients with meniscal tear and medial ME through a mini-incision with three interconnecting suture anchors (Fig. 2a), and found that postoperative ME was significantly reduced. Koga et al. [77] performed arthroscopic centralization of nine extruded lateral menisci with the use of suture anchors, and ME was significantly reduced in 2 years of follow-up.

Several other techniques of meniscal centralization with suture anchors as well as with transtibial tunnel, with or without prior meniscal release from its capsular attachments, have also been described in technical notes (Fig. 2b, c) [78–82]. However, to date there is still no well-designed clinical trial available to ascertain the effectiveness of centralization in reducing ME.

### Anterior cruciate ligament reconstruction

The ACL is the main restrictor in the anterior tibial translation, while the posterior part of the medial meniscus (MM) acts as a secondary stabilizer [83, 84]. The lateral meniscus (LM) is a secondary stabilizer in the knee joint during pivot-shift loading [85]. The goal of the anatomic reconstruction of the ACL rupture is to replicate the normal knee anatomy. However, the incidence of severe knee OA is much higher in patients who have been subjected to ACLR than individuals with intact ACL [86, 87]. It has been shown from studies that the risk for TKA after ACLR is seven times greater than in normal individuals [88].

The incidence of meniscal tears associated with ACL injury is as high as 79%, with the LM being affected more often than the MM in acute situations [89]. It is known that ACL and meniscal function cannot be fully restored even after surgical repair [86, 90]. To the best of our knowledge, the exact clinical significance of ME after ACLR has not yet been clearly elucidated, although efforts are continuously being made to minimize this phenomenon. ME depends largely on the severity and type of tear [87, 89, 91]. After ACLR, extrusion of the ΜΜ is more frequently seen than that of the LM. Moreover, extrusion of the LM does not improve after ACLR unless the meniscal tear is repaired [87, 92]. In addition, when there is ACL rupture and the preoperative extrusion of the LM is 1.1 mm, the surgeon should consider the possibility of a complete posterior root tear (PRT) of the LM. This value is identified as the optimal cutoff point for a complete PRT of the LM and has 100% sensitivity and 83% specificity [92]. In a recent study by Tsujii et al., it was shown that after concurrent ACLR and meniscal repair, the lateral extrusion of the LM was correlated with its healing status. If the repaired meniscus failed to heal, lateral extrusion progressed significantly [92]. However, its posterior extrusion progressed significantly regardless of the healing status [90]. The mechanical damage of the meniscus caused by extrusion seems to change the loading-bearing capacity of the tibiofemoral compartments, leading to the destruction of the articular cartilage as well the subchondral bone, ultimately contributing to the development or progression of OA [87, 89, 92].

An inappropriate tibial tunnel may also cause extrusion of the LM. A more posterolateral placement of the tibial tunnel aperture may induce extrusion of the LM following ACLR. The primary site of destruction is the soft tissue fibers which couple the deep layers of the ACL to the LM and the LM to the tibial plateau. These fibers specifically act on the LM anterior root attachment itself, explaining the reduction in the ultimate failure of the anterior root strength of the LM with disruption of these fibers [87, 93]. A novel technique has been developed based on a measurement grid in order to determine the precise aperture of the tibial tunnel. According to this technique, a more posterolateral position of the tibial tunnel aperture within the ACL footprint reduces the RTD (reference point to tibial tunnel distance) and increases the extrusion of the LM following ACLR [93].

Comparative studies between normal knees and knees with ACL rupture demonstrated that the posterior extrusion of the MM during knee flexion is almost the same [83, 94]. It was also shown that after ACLR, without concomitant tears of the menisci, the anteroposterior and radial extrusion of the MM tended to increase compared with preoperative levels, but all measurements remained below the threshold value of 3 mm [24, 44, 95]. These findings suggest that the tension of the graft and the excessive external rotation of the tibia are also factors that can affect the condition of the MM. Therefore, ACL rupture alone cannot cause the posterior extrusion of the MM, and the ACLR alone does not directly cause distinct damage to the MM. As a result, post-traumatic transposition and degeneration of the MM may not be completely prevented by ACLR alone. However, further investigation is needed to evaluate the effect of the ACLR on the restoration of the MM function [88].

Degenerative lesions of the knee joint are more commonly present during concurrent ACLR with meniscectomy than with intact or repaired menisci. The concurrent ACLR with repair of the menisci has shown good clinical and functional results and less risk of reoperation during the follow-up periods [83, 87]. However, over a long-term period, the degenerative changes in the articular cartilage worsened, especially in the lateral tibial plateau, regardless of the healing status of the meniscus [85, 87, 90]. Furthermore, Lee et al. (ACLR + LM tears left in situ) showed narrowing of 0.33 mm of the lateral joint space at 3 years postoperatively, and Shelbourne et al. (ACLR + LM tears left in situ) showed narrowing of 1.0 mm at 10-year follow-up [96, 97]. Tsujii et al. (ACLR + LM tear repair) demonstrated that the narrowing was only 0.04 mm at 3.4 years postoperatively, whereas another study showed widening of 0.1 mm at 3.5 years postoperatively [85, 90].

Although there is a need for longer follow-up periods, the currently available data suggest a benefit of concomitant ACLR with the repair of longitudinal, radial, posterior horn and posterior root tears of the menisci, because the combination can even better reduce their shape and extrusion [92, 98–100]. Winkler et al. showed that the difference in the extrusion of the LM between a healthy knee and a knee with combined injury was not statistically significant. Based on these findings, they concluded that the all-inside repair of the radial tear of the LM could help maintain its dynamic behavior, thus reducing the biomechanical stress exerted on the adjacent cartilage [89]. Another promising surgical approach for reducing the ME is the arthroscopic centralization of the extruded meniscus at the point where the capsule adjacent to the meniscus is repaired at the border of the tibial plateau [98, 101]. There is no established optimal surgical technique at present for restoring ME to normal values, and thus there will be a strong need for more effective and refined techniques.

### Meniscal allograft transplantation

To address the problems of patients with meniscectomy, efforts are constantly being made to develop techniques that aim at meniscal regeneration or development of meniscal scaffolds. MAT is a potential surgical procedure for young, active patients with symptomatic meniscal insufficiency that does not respond to conservative treatment [102, 103]. The early results of MAT are encouraging, as pain relief and improved knee function are reported [15–17, 62, 104–109]. In fact, MAT supporters claim that it restores the biomechanical properties of the endogenous meniscus, prevents or slows cartilage damage, especially if the MAT is performed immediately, and improves long-term outcomes [17, 110, 111].

Most authors agree that the transplanted menisci are extruded more frequently than endogenous menisci [15, 102, 105]. It should be highlighted that the clinical significance of meniscal allograft extrusion has not yet been clearly elucidated, since it is difficult to draw firm conclusions. For instance, an oversized allograft achieves adequate coverage of the tibial plateau while exhibiting a large amount of extrusion, whereas the opposite applies for undersized or shrunken allografts [112]. Meniscal allograft extrusion causes incongruity with the femoral condyle, and therefore the allograft cannot perform normal load transmission and shock absorption. This condition may lead to increased cartilage degeneration, progressive OA and allograft failure, as mentioned in several biomechanical studies [17, 111]. Moreover, it has been shown that allograft extrusion does not correlate with clinical outcomes and tends to be stable postoperatively during follow-up [62, 108, 113]. In addition, it has been shown that after lateral MAT, the extrusion is correlated with the progression of joint space narrowing (JSN) during long-term follow-up, while after medial MAT, JSN is observed during medium-term follow-up [114]. Finally, meniscal allograft extrusion is also associated with subchondral bone marrow lesions and cysts [2, 115].

MAT is not a simple surgical procedure, has a high learning curve, and if it is not performed under certain indications (Table 1) it fails, leaving both the surgeon and the patient dissatisfied [106, 109]. For these reasons, certain contraindications have been introduced (Table 2) [102, 103, 105–109]. Despite the advances in surgical techniques and the improved methods for the estimation of the implant size, MAT is successful in only up to 75% of patients [2, 16, 109]. There is no consensus whether medial or lateral MAT is superior in terms of medium-term (5–10 years) and long-term (> 10 years) survival. In a recent meta-analysis, the percentages of medium-term and long-term survival after medial MAT were 85.8% and 52.6%, respectively, whereas after lateral MAT they were 89.2% and 56.6%, respectively [116]. The survival of the graft depends substantially on the condition of the articular cartilage [117–119].

**Table 1:** Indications of meniscal allograft transplantation [106, 109]

1. Previous subtotal or total meniscectomy with persistent pain and swelling that does not respond to conservative treatment
2. Age ≤ 60 years (*Lee-2020*) < 55 years (*Kim-2017, Kim-2018*) < 50 years (*Jang-2011*) < 45 years (*Ha-2010, Ha-2014*)
3. Normal axis of the lower limb as shown in the scanogram
4. Absence of knee instability
5. Damage of the articular cartilage up to grade 2 according to the Outerbridge classification
6. Knee OA grade ≤ 2 according to the Kellgren–Lawrence or Ahlbäck classification*

*OA* osteoarthritis

*Localized articular cartilage degeneration of stage 3 or 4, which is limited to the area covered by the meniscus, is not considered a contraindication for meniscal transplantation (*Kim-2017, Kim-2018, Lee-2020*)

**Table 2:** Contraindications for meniscal allograft transplantation [102, 103, 105–109]

1. Age > 60 years
2. Poor axial alignment of the lower limb (varus/valgus > 3^ο^ or 5^ο^)
3. Immature skeleton
4. Knee OA of grade ≥ 3 according to the Kellgren–Lawrence or Ahlbäck classification
5. Diffuse degeneration of the articular cartilage
6. Knee instability
7. BMI > 35
8. Inflammatory joint disease
9. Disease of the synovial membrane
10. Recent septic arthritis or untreated septic arthritis
11. Metabolic disorders or the presence of crystals
12. Previous osteotomy for mechanical axis correction
13. ACL insufficiency

*OA* osteoarthritis, *BMI* body mass index, *ACL* anterior cruciate ligament

Several preoperative and intraoperative factors (Table 3) are associated with the absolute value of allograft extrusion [16, 102, 106, 109, 120]. This suggests that if patients with minimal degeneration and accurate indications undergo MAT with a well-placed graft, the graft extrusion will be minimal and the results will remain unchanged. However, evaluation by absolute value alone may be limited due to differences in implant size, bone and cartilage condition, and individual patient characteristics. Therefore, the relative values may be significant [17].

**Table 3:** Risk factors for/causes of meniscal allograft extrusion [16, 102, 106, 109, 120]

1. Size mismatch between the affected joint surface and the allograft
2. Excessive peripheral suture tension
3. Nonanatomical placement of the allograft
4. Pre-existing osteophytes on the tibial plateau
5. Loss of fixation of the anterior and posterior horns
6. Non-repair of meniscotibial ligaments and popliteomeniscal fascicles
7. Bony inclination of the allograft
8. Position of the bone bridge
9. Fixation of the allograft
10. Recipient–donor mismatch

Many techniques have been described for meniscal transplantation, but the most commonly used are (a) the suture-only technique, (b) bone-plug technique, (c) keyhole technique and (d) bridge-in-slot technique [102, 104, 111]. The results as to which technique is superior to the others remain controversial. Most transplantations have been performed with the bone-plug technique because previous studies have shown its superiority in load-bearing function, resistance to hoop stresses, similar contact mechanics to endogenous menisci and lower extrusion rates compared with the other techniques [109, 111]. On the other hand, the suture-only technique has its own advantages and is therefore chosen by many surgeons [104, 111].

Although meniscal allografts that are secured with the suture-only technique tend to show a greater degree of extrusion than the bony-fixation techniques, bony-fixation techniques still show high extrusion rates (25–50%). Therefore, improvements in surgical techniques are necessary [102, 109].

Several strategies have been proposed to reduce allograft extrusion. Although the ultimate cause is unknown, it is quite common to see a subluxated joint capsule in the symptomatic meniscectomized compartment. This is probably why capsulodesis is so effective in minimizing extrusion. Another successful strategy is osteophyte resection. Osteophyte removal > 2 mm at the edge of the lateral tibial plateau can reduce extrusion after lateral MAT [109, 121, 122]. Correct estimation of the allograft size is also important. It has been suggested that a 5–10% reduction of implant size from the estimated one reduces postoperative extrusion [2, 105, 123]. And finally, the proper anatomical placement of the allograft is essential. Nonanatomical placement of the meniscal allograft > 5 mm relative to the endogenous meniscus adversely affects the mechanical contacts of the knee, which may affect the allograft's ability to prevent articular cartilage degeneration [16, 124]. Improper positioning of the posterior horn causes a similar deviation to the anterior horn. Therefore, proper placement of the posterior horn is critical to the anatomical repair of the meniscus [125–127].

## Conclusion

**Fig. 3:**
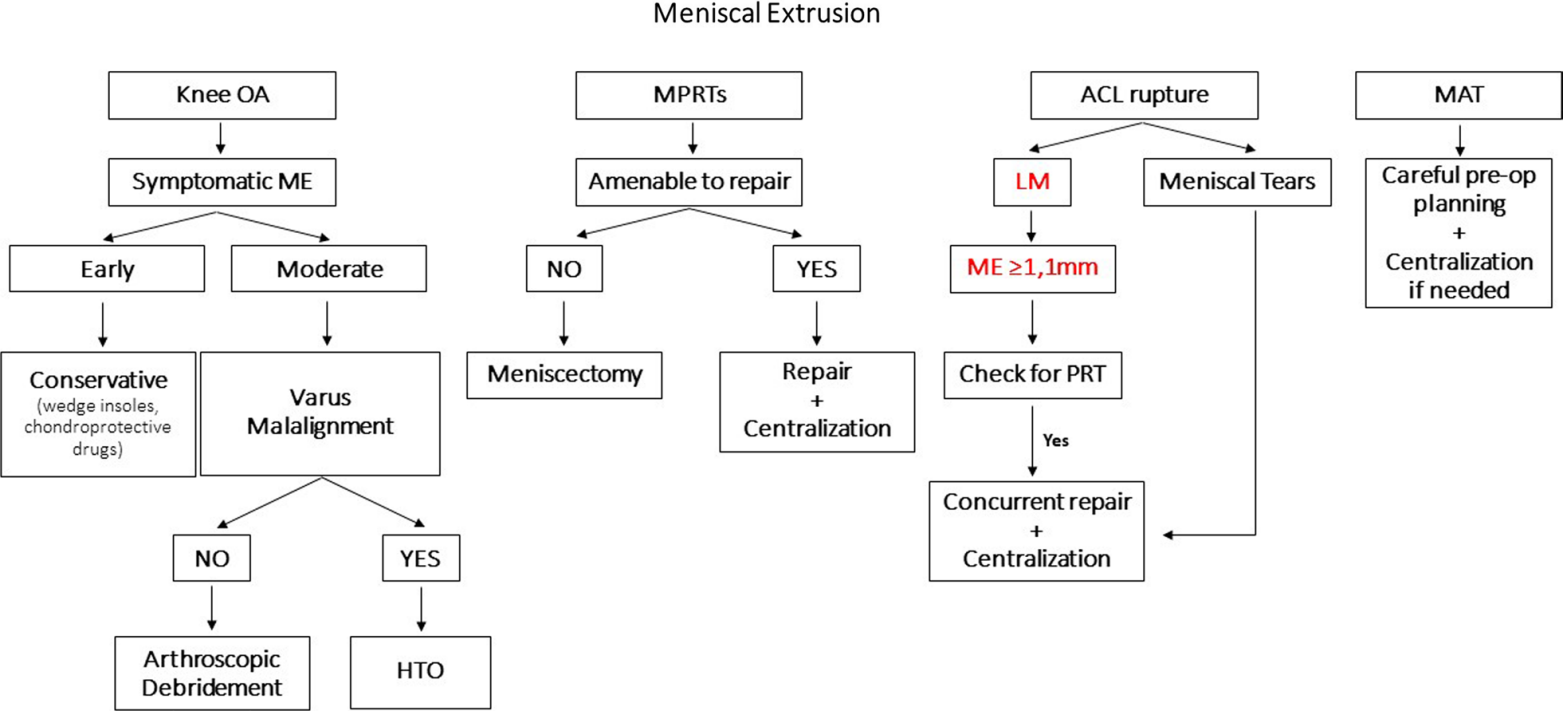
Proposed treatment algorithm for meniscal extrusion (ME) in different knee pathologies (*OA* osteoarthritis, *ME* meniscal extrusion, *HTO* high tibial osteotomy, *MPRTs* meniscal posterior root tears, *ACL* anterior cruciate ligament reconstruction, *LM* lateral meniscus, *PRT* posterior root tear, *MAT* meniscal allograft transplantation)

ME is found in several knee pathologies and is well correlated with rapid knee OA progression as well as increased morbidity. Therefore, care should be taken to manage it appropriately and successfully reduce it. Although in mild/moderate knee OA there is still no specific treatment targeting ME, the established conservative and operative treatment modalities seem equally successful in delaying the progression of OA and improving symptoms when ME is also present. Future research should focus on the role of additional centralization of the meniscus during arthroscopic debridement for the treatment of moderate knee OA. Although it has never been reported, it is worth researching, as it could have a beneficial effect. MPRT should be repaired earlier than 3 months after diagnosis. Furthermore, supplementary centralization of the meniscus appears to be able to adequately reduce the ME, as it also addresses potential MTL lesions. However, more research with high-quality studies is needed in order to refine the technique and validate its long-term results in reducing ME. Post-ACLR ME is also a concern and should be prevented by repairing concurrent meniscal tears and centralizing the meniscus along with the ACLR. Finally, meniscal allografts often result in extrusion, thus potentially reducing the clinical benefits of transplantation. Careful preoperative planning for correct sizing and accurate placement of the allograft is critical for preventing ME. Moreover, centralization of the allograft can be performed to help stabilize it on the tibial plateau; however, there are not yet sufficient data in the literature to support its benefits. Finally, we provide a proposed treatment algorithm based on the information retrieved from the literature that could help guide clinicians in the management of ME on the aforementioned knee pathologies (Fig. 3).
